# *In vivo* Estimation of Breast Cancer Tissue Volume in Subcutaneous Xenotransplantation Mouse Models by Using a High-Sensitivity Fiber-Based Terahertz Scanning Imaging System

**DOI:** 10.3389/fgene.2021.700086

**Published:** 2021-09-27

**Authors:** Hua Chen, Juan Han, Dan Wang, Yu Zhang, Xiao Li, Xiaofeng Chen

**Affiliations:** ^1^School of Physics, Southeast University, Nanjing, China; ^2^The First Affiliated Hospital of Nanjing Medical University, Nanjing, China

**Keywords:** THz, imaging, mouse model, breast cancer, caner volume

## Abstract

Absorption contrast between the terahertz (THz) frequency range of fatty and cancer tissues allows cancer diagnosis by THz imaging. We successfully demonstrated the ability of THz imaging to measure small breast cancer volume in the subcutaneous xenotransplantation mouse models even without external comparison. We estimated the volume detection limitation of the fiber-based THz scanning imaging system using a highly sensitive cryogenic-temperature-operated Schottky diode detector to be smaller than 1 mm^3^, thus showing the potential application of this technique in preliminary early cancer diagnosis.

## Introduction

Terahertz (THz) wavelength is from 0.003 to 3.0 mm, which is longer than far-infrared and light wave, so the scattering in a biological tissue is greatly reduced and no harmful photoionization occurs for the low photon energy (Kindt and Schmuttenmaer, 1996). Meanwhile, THz waves are very sensitive to polar substances (Pedersen and Keiding, 1992; Wang et al., 2010; Yamada et al., 2014) and can provide better contrast for the biological tissue than x-ray. So far, researchers have detected various human cancers by using THz wave. For example, skin cancer has been the focus of THz imaging research in recent years. It has been confirmed by *in vivo* and *in vitro* models that THz has a high diagnostic rate for the boundary and depth of invasion of skin cancer (Woodward et al., 2002; Rahman et al., 2016). Pickwell et al. (2005) measured the THz refractive index and absorptivity of normal tissues and cancer tissues of 10 patients with basal cell carcinoma and showed that the absorption characteristics of cancer tissues were significantly different from those of healthy tissues; this contrast between the two tissues proved that THz imaging can be used as a non-invasive diagnostic tool for skin cancer. Fitzgerald et al. (2006) analyzed the THz images of isolated breast cancer tissues and compared the imaging edge with pathological examination results. Reese (Reid et al., 2011) and other researchers have studied the THz images of freshly resected colorectal cancer tissues and found that normal tissues have a good contrast with cancer tissues and it is possible to detect cancer in esophagus, colon, bladder, prostate, and other deep tissues by THz endoscopic imaging equipment (Wang and Mittleman, 2004).

Breast cancer is the second most common cancer affecting women and accounts for 23% of all cancer cases. Moreover, it is also the main cause of cancer death for females, and the mortality rate is 14% of the all cancer deaths (Jemal et al., 2011). Recently, several preliminary clinical studies have reported that the THz absorption contrast method could be used to diagnose breast tumors from normal tissues (Fitzgerald et al., 2006; Ashworth et al., 2009; Chen et al., 2011a,b; Bowman et al., 2017a,b, 2018; Chavez et al., 2018), and the contrast is induced by water content and cancer-induced structure change (Ashworth et al., 2009; Chen et al., 2011a,b). In our previous study, we not only demonstrated that THz wave can clearly identify breast cancer tissue without any other H&E staining (Bowman et al., 2017b), but also realize early detection breast cancer in the nude mice (Chen et al., 2011a). However, the detection capability is limited to tissues thinner than 5 mm (Chen et al., 2011a), which is too thin compared to the thickness of an actual female breast under magnetic resonance imaging or x-ray (>5 cm), thus limiting further clinical applications. In this study, the capability was improved to 8 cm by applying a high-sensitive cryogenic-temperature-operated Schottky diode detector to the fiber-based THz scanning imaging system. Using this technique, we realized *in vivo* early breast cancer detection in a subcutaneous xenotransplantation mouse model without any external comparison, and even estimated the detection limit of the THz imaging system to be smaller than 1 mm^3^, which is a great advantage compared to the current detection limit of x-ray mammography (2 mm diameter).

## Experimental

### Setup of the Terahertz Imaging System

**FIGURE 1:**
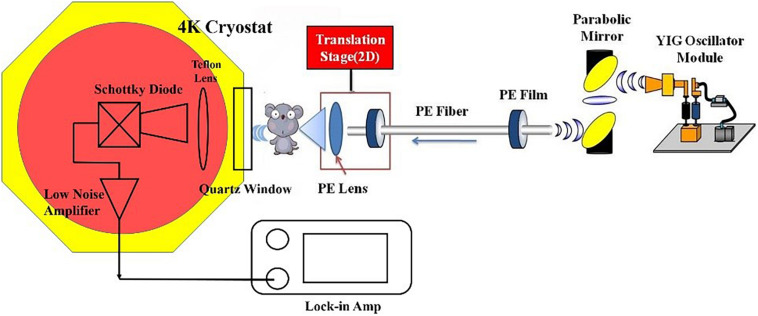
Schematic picture of the imaging system. Fiber parameters: diameter: 600 μm; length: 40 cm; attenuation coefficient: 10^–3^ cm^–1^.

The results of *ex vivo* THz spectroscopy of thin breast tissue sections (Fitzgerald et al., 2006; Bowman et al., 2017b) revealed that high tissue absorption leads to low penetration depth, which makes transmission imaging difficult. However, the THz absorption of the breast tissue decreases at lower frequency, so we use 108 GHz frequency for *in vivo* imaging. A schematic picture of the fiber-based THz imaging system used in this study is shown in Figure 1. The parameters of polyethylene (PE) fibers (Chen et al., 2006, 2007; Lu et al., 2008) and the working principle of the system remains unchanged from those described in our previous system (Chen et al., 2011a). Briefly, the THz wave is radiated from a YIG oscillator module, and then the THz wave is collected by a pair of off-axis parabolic mirrors and focused into the PE sub-wavelength fiber with a diameter of 600 μm and a length of 45 cm (Chen et al., 2011a). Finally, the THz wave coupling by TE fiber is focused by a PE lens onto the sample and then the transmitted power is detected by the detector. To improve detection sensitivity, we introduced a cryogenic-temperature-operated Schottky diode detector with a working temperature of approximately 4 K. Cooling the Schottky diode detector reduces noise significantly, thus enhancing the sensitivity to 10^–13^ W/Hz with the same dynamic range and response time. Finally, a lock-in amplifier will analyze the collected signals. The image is obtained by two-dimensional (*X*–*Y*) direct scanning of the output end of the fiber with an imaging time of less than 1 min. The results show the signal-to-noise ratio of the imaging system to be about 10^8^:1, which is improved about 10^3^ times compared to our previous imaging system (Chen et al., 2011a).

### Mouse Treatment

This work is approved by the Institutional Animal Care and Use Committee of Southeast University and Nanjing Medical University (No. 3207027381). We purchased 4- to 6-month-old female BALB/cAnN.Cg-Foxnlnu/CrlNarl mice, an immune inhibited laboratory mouse strain unable to reject breast cancer cell injection and fatty tissue xenograft from another species, from Slac Laboratory Animal, Shanghai, China.

To induce breast cancer, we directly implanted 0.3 ml of MDA MB 231 breast cancer cells into the dermis layer of the mouse skin. The cancer cells were cultivated in L-15 with 10% fetal bovine serum and 1% antibiotics to a cell concentration of 5 × 10^7^ per milliliter of culture media. After injecting the cancer cells, we immediately marked the injection area, kept the mice warm around 36°C, and restored them to health. On the seventh day, we implanted mouse fatty tissue to embed the breast cancer cells. The implanted or *ex vivo* measured fatty tissue was aspirated from 12-week-old female B6.V-Lepob/J mice and rinsed thrice in the transport medium [NaCl 0.9% (w/v), glucose 56 mM, HEPES 25 mM, and PSA 10 ml (pH 7.4)]. The cancer cells and fatty tissue implantations as well as *in vivo* THz imaging were conducted after anesthetizing the mice by injecting ketamine-xylazine (50 + 15 mg/kg) intraperitoneally. THz imaging was conducted 7 days after fatty tissue implantation.

### THz Absorption Spectra of Mouse Tissues

**FIGURE 2:**
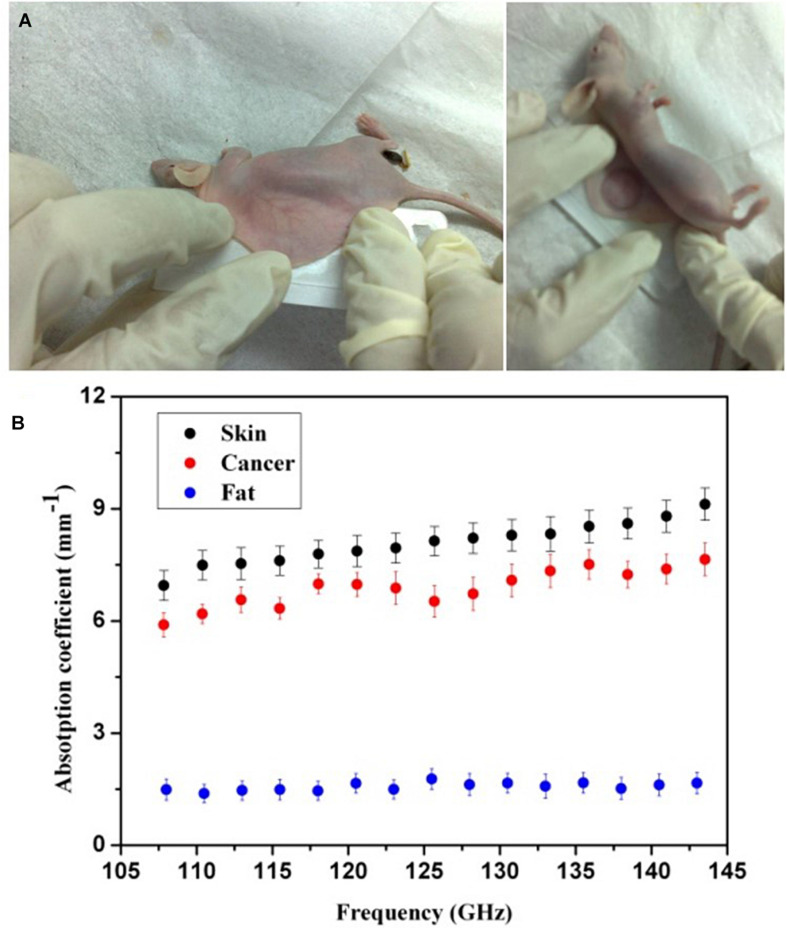
**(A)** Photos of a mouse processing. **(B)** Absorption spectra of mouse skin and fatty and breast cancer tissues (black, blue, and red solid circles, respectively). The error bars represent the standard deviation of the mean; *n* = 20.

We first *in vivo* measured the mouse skin, fatty tissue, and breast cancer tissue by THz absorption spectroscopy at 108–143 GHz. To extract the properties of the constituent tissue types, THz absorbance (α) was averaged linearly by assuming that any reflections and scattering caused by heterogeneities within samples were negligible. The absorbance was calculated according to the Beer-Lambert law α = ln(*I*_*s*_*/I*_*b*_)/*d*, where *I*_*s*_ is the transmitted power of the THz wave through samples, *I*_*b*_ is the background (transmission power of THz wave through the cover glass), and *d* is the thickness of tissues. As shown in Figure 2A, after anesthetizing the mouse, we sandwiched the embedded dorsal area with two cover glasses. Then, the THz absorption spectra were measured by the YIG oscillator module and Schottky diode detector mentioned in section “Setup of THz Imaging System.” The corresponding absorption coefficients were calculated from the measurements in 20 mice, which is shown in Figure 2B. It has been clearly found that THz absorption spectra can differentiate between fatty and cancer tissues and the absorption coefficients of cancer tissues are much higher than those of the fatty tissues. As the water content of breast cancer tissues is higher than normal tissues, we believe that the water content in tissues may be the most related and dominant factor for the absorption contrast (Chen et al., 2015). Meanwhile, we found that the absorption coefficients of skin and cancer tissue were similar. Considering that skin thickness is relatively uniform and will not vary with time significantly, we calibrated the attenuation due to skin as a uniform and position-independent attenuation background. Moreover, considering the sensitivity of the cryogenic-temperature-operated detection system and absorption coefficients of mouse skin and fatty and breast cancer tissues, we estimated that the penetration capability of our system can be improved to 8 cm, which is similar to the average breast thickness in Asian females.

## Results

**FIGURE 3:**
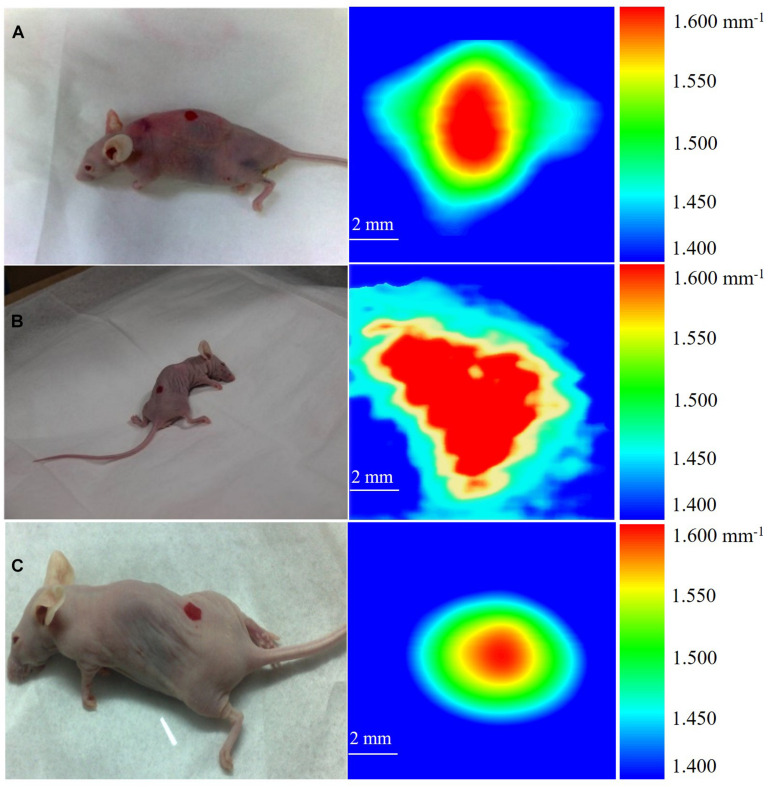
THz images of breast cancer in three mice. **(A)** The cancer in mouse is about 0.480 mm^3^. **(B)** The cancer in mouse is about 0.853 mm^3^. **(C)** The cancer in mouse is about 0.704 mm^3^.

After the cancer cell injection, on the 7th day, we anesthetized the mouse and implanted mouse fatty tissue to embed the cancer cells. Starting from the 14th day, we measured the cancer implanted area (marked as red area in the picture of Figure 3) by THz imaging daily. The mouse was anesthetized and the dorsal cancer area was sandwiched by two cover glasses. Finally, once the scanning completed, mice were monitored, kept warm to 36°C, and allowed to wake up naturally. For further studies on estimating the breast cancer size in the mouse model, we first tested the sensitivity of the THz imaging system with 10 mice, and the limitation was investigated in three mice.

Figure 3 shows the 10 × 10 mm^2^ THz images of three mice acquired on the 14th day after cancer cell implantation. During the imaging process, each mouse was scanned three times and the images were presented in the form of the mean absorption coefficient (α). We calibrated the attenuation due to skin as a uniform and position-independent attenuation background. The scanned images show that the high absorption of breast cancer tissue provides endogenous contrast under THz imaging, making it easy to distinguish from the background absorption. We defined the color bar by absorption coefficient α from 1.400 mm^–1^ to 1.600 mm^–1^. The background of the image, shown as blue color, is defined as 1.400 mm^–1^ < α < 1.450 mm^–1^, corresponding to the absorption coefficient of fatty tissue (according to Figure 2). The absorption coefficients of the sandwiched tissues induced with early cancer development is 1.450 mm^–1^ < α < 1.600 mm^–1^, while 1.600 mm^–1^ is the maximum absorption coefficient and is shown as red color. Since early cancer development differs individually, the absorption change Δα will be different for each individual, and the absorption change Δα for these three tested mice was 0.090, 0.160, and 0.132 mm^–1^, respectively.

According to the concept of cell absorption cross (σ), we estimated tumor volume in these three mice. σ is defined as: σ = α/*N* = α × *V*_*cell*_, where *N* is the number of absorbing cells per unit volume and *V*_*cell*_ is the volume of a single cancer cell. The development of cancer cells embedded in fat then induced Δα and the corresponding cancer cell density *N*’ was described as *N*’ = Δα/σ = Δα/(α × *V*_*cell*_). Finally, the volume of the total cancer tissue V was evaluated. Through the THz absorption spectra shown in Figure 3, we calibrated the value of σ. As shown in Figure 3, the measured absorption changes 0.090, 0.160, and 0.132 mm^–1^ in the three mice correspond to *V* = 0.480, 0.853, and 0.704 mm^3^, respectively, while the sensitivity of x-ray mammography depends on breast density (Nass et al., 2001) and the detection limitation is as small as 2 mm in diameter (Onuigbo et al., 2001) currently.

## Discussion

According to our previous study on human breast cancer, we proved that THz imaging can clearly diagnose breast cancer tissues (Chen et al., 2011b) and detect cancer volume (Chen et al., 2011a). However, the detection capability of the imaging system is far from clinical application, for the reason that the detection thickness of the former system is smaller than 5 mm (Chen et al., 2011a). In this study, we successfully improved the capability to 8 cm and clinical application would become possible compared to the thickness of an actual female breast. In order to further demonstrate the potential clinical application of THz imaging in the detection of small breast cancer tissue volume, we conducted this study in mouse models. The results show that THz imaging has high sensitivity and potential for non-invasive early cancer detection without exogenous contrast. In this work, we did not consider human breast fibrous tissue because the available subcutaneous xenotransplantation animal models prevent us from implanting fibrous tissue to simulate real females breast conditions. However, the THz absorption spectra can distinguish breast cancer tissue from fibrous tissue very well (Fitzgerald et al., 2006; Ashworth et al., 2009; Bowman et al., 2017b). The future potential, specificity, and penetration ability for *in vivo* imaging in humans needs to be studied.

## Conclusion

The fiber-based THz scanning imaging system based on cryogenic detection system was used to study human breast cancer tissue volume in the mouse model. Results show that THz imaging can not only monitor cancer development in real time but also identify small cancer tissue volume, and all the measurements are conducted without the need of exogenous contrast. Through calculation, we found that this method may be used to detect cancer tissue volume smaller than 1 mm^3^, which is highly advantageous compared to the current detection limit (2 mm) of x-ray mammography. This non-invasive and non-ionizing imaging method has a potential application to breast cancer volume detection.

## Data Availability Statement

The raw data supporting the conclusions of this article will be made available by the authors, without undue reservation.

## Ethics Statement

The animal study was reviewed and approved by the Institutional Animal Care and Use Committee of Southeast University and Nanjing Medical University (No. 3207027381).

## Author Contributions

HC, JH, and DW conducted this study and clinical trials. DW, YZ, and JH conducted the experiments. XC and XL analyzed the data. HC wrote the main manuscript text. All authors contributed to the article and approved the submitted version.

## Conflict of Interest

The authors declare that the research was conducted in the absence of any commercial or financial relationships that could be construed as a potential conflict of interest.

## Publisher’s Note

All claims expressed in this article are solely those of the authors and do not necessarily represent those of their affiliated organizations, or those of the publisher, the editors and the reviewers. Any product that may be evaluated in this article, or claim that may be made by its manufacturer, is not guaranteed or endorsed by the publisher.
